# Isolated Intraconal Meningioma

**DOI:** 10.18502/jovr.v16i4.9759

**Published:** 2021-10-25

**Authors:** Mohammad Taher Rajabi, Kasra Cheraqpour, S. Saeed Mohammadi, Mohammad Veshagh, Seyedeh Zahra Poursayed Lazarjani, Farideh Hosseinzadeh, Fahimeh Asadi Amoli, Simindokht Hosseini

**Affiliations:** ^1^Eye Research Center, Farabi Eye Hospital, Tehran University of Medical Sciences, Tehran, Iran; ^2^Ophthalmic Research Center, Research Institute for Ophthalmology and Vision Science, Labbafinejad Medical Center, Shahid Beheshti University of Medical Sciences, Tehran, Iran; ^3^Department of Ophthalmology, Guilan University of Medical Sciences, Rasht, Iran; ^4^ENT and Head & Neck Research Center, The Five Senses Health Institute, Iran University of Medical Sciences, Tehran, Iran; ^5^Pathology Department, Farabi Eye Hospital, Tehran University of Medical Sciences, Tehran, Iran; ^6^Department of Ophthalmology, Abadan University of Medical Sciences, Abadan, Iran

**Keywords:** Ectopic Meningioma, Intraconal Meningioma, Orbital Meningioma, Primary Meningioma

## Abstract

**Purpose:**

To report a rare case of isolated intraconal meningioma.

**Case Report:**

A 24-year-old woman presented with painless proptosis in her left eye which started and progressed during her pregnancy about 10 months ago. Hertel exophthalomometry revealed anterior displacement of the globe with 4 mm of proptosis which was remarkable. Magnetic resonance imaging (MRI) demonstrated an intraconal circumscribed oval-shaped mass with hypointense signals on T1-weighted images and hyperintense signals on T2-weighted images, mimicking cavernous hemangioma. This mass, however, was free of any connections to optic nerve or bones. Due to the imaging characteristics, more prevalent diagnoses like cavernous hemangioma were placed on the top of the differential diagnoses list. However, during the surgical excision, the tumor’s consistency and gross features were not compatible with cavernous hemangioma. The pathologic findings instead determined meningotheliomatous meningioma, a very rare condition, which was far from our expectations prior to the surgery.

**Conclusion:**

Ectopic orbital meningiomas are rare tumors that are not easily diagnosed without postoperative histopathology. Despite its low prevalence, they should be considered in the differential diagnosis list of intraconal masses with hypointense signals on T1-weighted images and hyperintense signals on T2-weighted images.

##  INTRODUCTION

Orbital meningiomas account for 0.4–2% of all meningiomatous tumors.^[1]^ These lesions are further subdivided into three classifications. The first classification is primary optic nerve sheath meningiomas (ONSM) originating from the arachnoid layer of the optic nerve ( < 30% of cases). The second classification is secondary ONSM arising from the sphenoid wing (e.g., intracranial meningiomas accounting for < 70% of orbital meningiomas). The last rare group is ectopic meningiomas which are free from any connections to the optic nerve or intracranial meninges ( < 1%).^[2,3,4,5,6,7,8,9]^ Ectopic orbital meningioma is usually located on the medial part of the orbit.^[10]^ This uncommon entity of meningiomas usually reveals as a well-circumscribed mass but an ill-defined border does not rule out this type of tumor.^[10]^ Herein, we report a rare case of ectopic (isolated) intraconal meningioma.

##  CASE REPORT

A 24-year-old woman presented with painless proptosis in her left eye, which started and progressed during her pregnancy about 10 months ago. Her uncorrected- and best-corrected visual acuity (UCVA and BCVA) were 20/25 and 20/20, respectively. In addition, the relative afferent pupillary defect (RAPD) in the left eye was negative. While the Hertel exophthalmometry revealed an anterior displacement of the globe with 4 mm of proptosis, the fundoscopy showed a left optic disc edema. Other slit-lamp examinations were normal.

**Figure 1:**
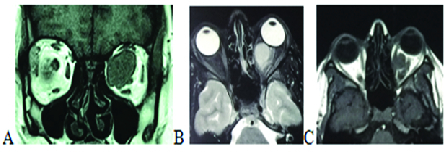
Orbital Magnetic Resonance Imaging (MRI). (A) Coronal image illustrating a superonasal intraconal mass on T1-weighted images which is separated from optic nerve. (B & C) Axial image illustrates a hypointense oval-shaped intraconal mass on T1-weighted images and hyperintense on T2-weighted images.

Magnetic resonance imaging (MRI) demonstrated an intraconal circumscribed oval-shaped mass with hypointense signals on T1-weighted images and hyperintense signals on T2-weighted images [Figure 1] mimicking cavernous hemangioma.

As a consequence, the patient underwent uncomplicated superomedial orbitotomy which resulted in the removal of a 3 × 1 × 0.5 cm necrotic white mass without any bleeding and which was also free of connections to the optic nerve sheath.

**Figure 2:**
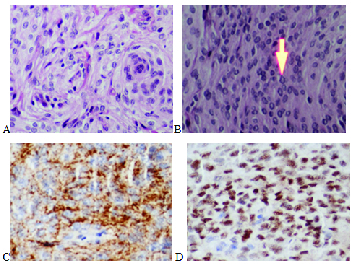
Histopathology. (A) Hematoxylin and Eosin (H&E) staining illustrating meningioma, tumoral cells with syncytial and whirling arrangement. (B) H&E staining showing intranuclear pseudo-inclusions. (C & D) Immunohistochemistry illustrating positive staining for Epithelial Membrane Antigen (EMA) (cytoplasmic), Progesterone Receptor (PR) (nuclear), respectively.

A histopathological examination showed tumoral cells with syncytial and whirling arrangement, indistinct cell membranes, eosinophilic cytoplasma, and rather uniform nuclei. Some intranuclear pseudoinclusions were also present. Mitotic figures were rare. Immunohistochemistry revealed positive staining for epithelial membrane antigen (EMA) a progesterone receptor (PR) [Figure 2] and negative staining for S100, CD34, and BCL2. Ki67 showed proliferative activity in about 1–2% of tumor cells. As a result, a meningotheliomatous meningioma (WHO grade 1) diagnosis was made.

Postoperative radiotherapy was performed on the orbital tumor bed. After a one-year follow-up, no complications or changes in the patient's perimetry, visual acuity, and RAPD were detected.

##  DISCUSSION

When assessing an intraconal mass with hypointense signals on T1-weighted images and hyperintense signals on T2-weighted images, several differential diagnoses should be considered, such as cavernous hemangioma, schwannoma, lymphoma, and neurofibroma. In our case, the orbital mass was intraconal without connections to the optic nerve or bones. Due to the imaging features of the lesion, a more prevalent diagnosis, such as cavernous hemangioma was expected. However, during the surgical excision of the lesion, the tumor's consistency and gross features were different from that of a cavernous hemangioma and the pathological evaluation of the mass determined meningotheliomatous meningioma.

**Table 1:** Brief review on recent reports


**No. ^[Ref]^**	**Patient**	**History & Examination**	**VA**	**Imaging**	**Treatment**	**Outcome**
Gündüz et al^[10]^	56/F	*3 months of slowly progressive proptosis and eyelid swelling *5 mm of proptosis (OD)	20/20	*MRI: Ill-defined mass in the right superior orbit with isointense signals and respect to the orbital fat and cerebral gray matter on T1WI, hypointense signals on T2WI, and moderate contrast enhancement *CT scan: Superiorly located mass producing thinning of the overlying bone	Subtotal resection through superonasal orbitotomy + conventional external beam radiotherapy	*74 months F/U without recurrence *VA of CF at 2 meters due to radiation retinopathy
	27/M	*Slowly progressive proptosis over 6 months *12 mm of proptosis *Limitation on elevation and abduction *Conjunctival edema and injection over the LR muscle insertion (OS)	20/20	*MRI: Well-defined tumor laterally in the orbit with hypointense signals on T1WI, hyperintense signals on T2WI, and moderate contrast enhancement *CT scan: No connection to the bony orbit	Anterior orbitotomy via a superolateral approach resulted in resection of 70% of the tumor + intensity modulated radiotherapy	*At 24 months F/U, VA was 20/20, and there was 2 mm of residual proptosis
Decock et al^[3]^	66/M	*4 years of growing orbital mass protruding upper eyelid *A firm mass not adherent to bone or skin (OS)	20/20	*CT scan: Extensively calcified mass located at the anterior edge of the lacrimal fossa without hyperostosis or involvement of the adjacent orbital bone	Translid surgical approach	15 months of F/U without recurrence
Huang et al^[13]^	7/M	*5 months of proptosis, upper eyelid edema, and diplopia	1.2	*Coronal T1WI showed a superonasal mass. Axial T2WI showed an ill-defined and heterogeneous mass and adjacent MR. (Misdiagnosed as capillary hemangioma)	Complete surgical resection in all cases	No recurrence or diminution of vision in none of cases
	18/F	*24 months of proptosis, ptosis, upper eyelid edema, and diplopia	1.2	*Axial T1 showed an ill-defined and heterogeneous superonasal mass and adjacent MR (Misdiagnosed as capillary hemangioma)	
	31/M	*12 months of proptosis, upper eyelid edema, and diplopia	LP	*T1WI MRI was hypointense and T2WI MRI was hyperintense * Axial CT scan showed a well-defined intraconal mass adjacent to the anterior optic nerve (Misdiagnosed as cavernous hemangioma)	
	35/M	*72 months of proptosis, ptosis, upper eyelid edema, and diplopia	1.0	*Coronal T1 W1 showed the superonasal mass and no adjacent MR. Axial T1 W1 showed the ill-defined and heterogeneous superonasal mass (Misdiagnosed as eosinophilic granuloma)	
	56/M	*3 months of proptosis, ptosis, upper eyelid edema, and diplopia	1.0	*T1WI MRI was hypointense and T2WI MRI was hyperintense *Axial CT scan showed a well-defined intraconal lesion with a calcified mass. Optic nerve was compressed and dislocated but integrated into the structure (Undiagnosed)	
	52/F	*6 months of proptosis, ptosis, upper eyelid edema, and diplopia	0.5	*T1WI MRI was hypointense and T2WI MRI was hyperintense (in all 6 cases) (Misdiagnosed as neurofibromatosis)	
Pushker et al^[7]^	30/F	*18-month of proptosis *3 mm proptosis and limitation in elevation (OS)	20/20	*CT scan: Ill-defined, heterogenous enhancing soft tissue mass involving the left superior extraconal space + associated expansion and sclerosis of the left half of the frontal bone and roof of the left orbit with few ill-defined lytic lesions	Excision through the anterior orbitotomy via a sub-brow incision	*Recurrence after 8 months resulted in re-surgery *No diminution of vision and further recurrence over an 18-month period
	40/M	*2-year history of painless, progressive proptosis *6 mm of proptosis and limitation in elevation (OS)	20/20	*CT scan: Homogeneous well-defined, intensely enhancing soft tissue mass in the left superomedial orbit	Rupturing of mass during excision resulted in piecemeal removal	*Recurrence of the mass after 11 months resulted in re-surgery *No further recurrence or diminished vision over 2 years F/U
	9/M	*2.5-year history of progressive Proptosis *5 mm of proptosis with (OS)	20/20	*CT scan: Diffuse, mildly enhancing and associated with hyperplasia of the adjacent bone	Piecemeal removal	No diminished vision over 3 months
Tendler et al^[11]^	9/F	*Gradual painless swelling of the medial upper eyelid *2 mm of proptosis and a firm mobile palpable mass in the superior nasal orbit of the (OS)	20/25	*MRI: Extraconal enhancing mass in the left medial orbit with notable ethmoid sinus asymmetry	Excision + proton beam therapy and surgical debulking after recurrence	Not reported
VA, visual acuity; F, female; M, male; OD, right eye; OS, left eye; MRI, magnetic resonance imaging (MRI); CT scan, computed tomography scan; T1W1, T1-weighted image; T2W1, T2-weighted image; F/U, follow-up; CF, counting fingers; LP, light perception; LR, lateral rectus; MR, medial rectus				

As a result, our patient underwent an orbitotomy with a partial tumor resection due to its fragile nature. Systemic work up was normal and the patient was referred for adjunctive radiotherapy. Although recurrence is rare in cases of complete excision, it should be mentioned that incisional biopsy has been known to accelerate spreading and recurrence of the tumor.^[11]^

It should be mentioned that other possible diagnoses such as sclerosis or hyperplasia of the superior orbital rim or asymmetry of the sinuses^[7]^ were ruled out in our case.

Orbital meningiomas most commonly arise from the base of skull or optic nerve sheath while ectopic orbital meningiomas are extremely rare accounting for < 1% of cases. Many previous studies have reported ectopic orbital meningiomas being located along the medial wall and superonasal rim.^[10]^ Origin of ectopic meningiomas has always been debated. These tumors may originate from congenitally dislocated nests of meningothelial cells, regressed orbital meningoceles located within the orbit, or curiously associated with dislocated meningeal tissues into the orbit secondary to penetrating injury or trauma.^[10]^ Interestingly, several reports exist regarding extracranial or extradural meningiomas found in unusual sites such as the neck, skin, finger, lung, mediastinum, and adrenal gland.^[11]^

Lee Teak Tan et al reported a case of presumed ectopic orbital meningioma which was decidedly diagnosed as olfactory groove meningioma. As a consequence, he hypothesized that a number of the previously reported cases have had similar scenarios of misdiagnosis. Having said that, other distinguished researchers continue to consider ectopic meningioma as a distinct entity where the origin and existence of ectopic orbital meningioma is still being debated.^[12]^

To date, there have been few reports of intraconal ectopic meningioma cases. A brief review of some of the recent reports of ectopic meningioma cases and other related issues such as demographic data, history, imaging findings, treatment, and final outcomes are summarized in Table 1.

The unique aspect of this case which has not been reported in prior studies is the development of an ectopic meningioma during pregnancy. This coincidental discovery on the possible association between pregnancy and the development and enlargement of meningiomatous tumors currently has no precedence and hence no available supporting data. However, it is recommended that treatment of these tumors be executed to ensure prevention of focal aggression.^[11]^ As a consequence, future case would need to be monitored to ensure all avenues are investigated.

In summary, as ectopic orbital meningiomas are characteristically very rare tumors and are not easily diagnosed with orbital imaging because of similar resemblance to other intraorbital tumors, serious consideration should be made in ensuring execution of postoperative histopathology to determine the existence or absence of these low prevalent tumors. The possible occurrence of these meningiomas should also be considered in the differential diagnosis of intraconal masses with hypointense signals on T1-weighted images and hyperintense signals on T2-weighted images. This approach would ensure the accurate diagnosis of conditions and would encourage the appropriate course of treatment.

##  Financial Support and Sponsorship

Nil.

##  Conflicts of Interest

There are no conflicts of interest.
